# Disruption of *OsSEC3A* increases the content of salicylic acid and induces plant defense responses in rice

**DOI:** 10.1093/jxb/erx458

**Published:** 2017-12-30

**Authors:** Jin Ma, Jun Chen, Min Wang, Yulong Ren, Shuai Wang, Cailin Lei, Zhijun Cheng, Sodmergen

**Affiliations:** 1 Key Laboratory of Ministry of Education for Cell Proliferation and Differentiation, College of Life Sciences, Peking University, Beijing, China; 2 National Key Facility for Crop Gene Resources and Genetic Improvement, Institute of Crop Science, Chinese Academy of Agricultural Sciences, Beijing, China

**Keywords:** Defense responses, exocyst complex, lesion-mimic phenotype, *Oryza sativa*, pathogenesis, salicylic acid, SEC3A

## Abstract

The exocyst, an evolutionarily conserved octameric protein complex involved in exocytosis, has been reported to be involved in diverse aspects of morphogenesis in Arabidopsis. However, the molecular functions of such exocytotic molecules in rice are poorly understood. Here, we examined the molecular function of OsSEC3A, an important subunit of the exocyst complex in rice. The *OsSEC3A* gene is expressed in various organs, and OsSEC3A has the potential ability to participate in the exocyst complex by interacting with several other exocyst subunits. Disruption of *OsSEC3A* by CRISPR/Cas9 (clustered regularly interspaced short palindromic repeats/CRISPR-associated protein 9) caused dwarf stature and a lesion-mimic phenotype. The *Ossec3a* mutant exhibited enhanced defense responses, as shown by up-regulated transcript levels of pathogenesis- and salicylic acid synthesis-related genes, increased levels of salicylic acid, and enhanced resistance to the fungal pathogen *Magnaporthe oryzae*. Subcellular localization analysis demonstrated that OsSEC3A has a punctate distribution with the plasma membrane. In addition, OsSEC3A interacted with rice SNAP25-type t-SNARE protein OsSNAP32, which is involved in rice blast resistance, via the C-terminus and bound to phosphatidylinositol lipids, particularly phosphatidylinositol-3-phosphate, through its N-terminus. These findings uncover the novel function of rice exocyst subunit SEC3 in defense responses.

## Teaser

Rice exocyst subunit OsSEC3A interacts with OsSNAP32, a SNAP25-type SNARE protein, and disruption of *OsSEC3A* causes a lesion-mimic phenotype and enhanced defense response

## Introduction

Upon attacks by pathogens, host plant cells activate a wide range of defense responses. Endocytic and exocytic vesicle trafficking is required for diverse aspects of defense responses ranging from regulation of early signaling to deployment of immune responses at later stages (Robatzek, 2007; Žárský *et al.*, 2013; Inada and Ueda, 2014). Recently, plant soluble SNARE complexes, which are involved in vesicle trafficking during exocytosis, were shown to be involved in immune signaling. HvSNAP34, a SNAP25 (soluble *N*-ethylmaleimide-sensitive factor attachment protein 25)-type SNARE protein in barley, inhibits pathogen penetration into host cells and is required for resistance to powdery mildew (Collins *et al.*, 2003). In rice, the SNAP25-type SNARE protein OsSNAP32 is involved in rice blast resistance (Luo *et al.*, 2016). PEN1/SYP121, a Qa-SNARE protein in Arabidopsis, is believed to function by forming a ternary SNARE complex with SNAP33, vesicle-associated membrane protein (VAMP) 721, and VAMP722 (Kwon *et al.*, 2008).

Before SNARE-mediated membrane fusion, the exocyst mediates tethering of vesicles to the plasma membrane in the early stage of exocytosis (Guo *et al.*, 2000). The exocyst, an evolutionarily conserved protein complex composed of Sec3, Sec5, Sec6, Sec8, Sec10, Sec15, Exo70, and Exo84, was initially discovered in yeast (TerBush *et al.*, 1996; Liu and Guo, 2012). Yeast Sec3 localized to the bud tip independent of the actin cytoskeleton is a landmark protein of polarized exocytosis that is involved in recruitment of the other exocyst subunits residing in the cytoplasm to the plasma membrane (Finger *et al.*, 1998; Wiederkehr *et al.*, 2003). The polarized localization of Sec3 to the plasma membrane is mediated by its interaction with phosphatidylinositol 4,5-bisphosphate [PI(4,5)P_2_] in the inner leaflet of the plasma membrane in yeast and mammalian cells (He *et al.*, 2007; Liu *et al.*, 2007; Zhang *et al.*, 2008). In yeast, Sec3 is a downstream effector of Cdc42 and Rho; interaction of Sec3 with Cdc42 and Rho is important for its function in exocytosis and cell polarity (Guo *et al.*, 2001; Zhang *et al.*, 2008).

In terrestrial plants, the exocyst exists as a subcomplex that primarily functions in regulating polarized exocytosis during cell expansion (Žárský *et al.*, 2009). While budding yeast, fungi, and most animals have only a single copy of each exocyst subunit, expansion of exocyst subunit genes, especially *Exo70* genes, in plants has resulted in the presence of several copies of some exocyst subunits. For example, Arabidopsis has 23 *Exo70* genes, whereas rice (*Oryza sativa*) has 47 *Exo70* genes; the functions of these copies are largely unknown (Chong *et al.*, 2010). Corresponding to the complexity of plant exocyst subunits, orthologs of exocyst subunits display varying specialized developmental functions. *Sec5*, *Sec6*, *Sec8*, and *Sec15* subunits encoded by one or two copies of each gene function in pollen germination, pollen tube growth (Hála *et al.*, 2008), and secretory processes during cytokinesis (Fendrych *et al.*, 2010). *Exo70A1* in Arabidopsis has been well characterized and functions in specialized developmental processes (Synek *et al.*, 2006; Samuel *et al.*, 2009; Fendrych *et al.*, 2010; Kulich *et al.*, 2010; Drdova *et al.*, 2013). Few rice exocyst subunits have been studied in detail, although rice exocyst subunit *OsEXO70A1* is known to be required for normal vascular bundle differentiation and primary mineral nutrient assimilation (Tu *et al.*, 2015).

Recent reports demonstrated that some plant EXO70 proteins are involved in plant immunity. For example, Arabidopsis *Exo70B2* and *Exo70H1* are up-regulated in response to pathogens and necessary for immune responses (Pečenková *et al.*, 2011). EXO70B2 is a target of plant U-box-type ubiquitin ligase 22 and negatively regulates PAMP (pathogen-associated molecular pattern)-triggered responses (Stegmann *et al.*, 2012). Mutation of EXO70B1 leads to activation of a defense pathway with an intracellular immune system receptor TIR-NBS2 (Zhao *et al.*, 2015). In addition, EXO70B1 is involved in autophagy-related membrane trafficking to vacuoles (Kulich *et al.*, 2013). In *Nicotiana benthamiana*, exocyst subunit *SEC5* is required for secretion of pathogenesis-related protein PR-1 and plays a role in CRN2-induced cell death (Du *et al.*, 2015). Although little is known about the function of the exocyst complex in rice, *OsExo70F3* was reported to be involved in AVR-*Pii*-triggered immunity to *Magnaporthe oryzae* by forming an AVR-*Pii*–OsExo70F2/3 complex (Fujisaki *et al.*, 2015).

The role of exocyst subunit SEC3 in plants has been widely investigated. This subunit is required for root hair elongation, embryogenesis, and pollen germination (Elias *et al.*, 2003; Wen *et al.*, 2005; Zhang *et al.*, 2013; Bloch *et al.*, 2016). However, the function of SEC3 in rice has not been elucidated. Although SEC3 has been reported to have molecular characteristics similar to those of EXO70, namely anchoring the exocyst to the plasma membrane (Wiederkehr *et al.*, 2003), there is no evidence linking SEC3 with plant immunity. In this study, we confirmed the ability of OsSEC3A to participate in the exocyst complex and obtained *OsSEC3A* mutants (*Ossec3a*) using CRISPR/Cas9 (clustered regularly interspaced short palindromic repeats/CRISPR-associated protein 9) technology. Our results showed that lesion spots that resembled the phenotype of lesion-mimic mutants (LMMs) appeared in *Ossec3a*, along with induced cell death and defense responses. As clues to the mechanism underlying the observed defense responses, we show that OsSEC3A interacted with rice SNAP25-type SNARE protein OsSNAP32 and phosphatidylinositol-3-phosphate [PI(3)P], which are involved in plant disease resistance, through the C-terminus and N-terminus, respectively. These results provide new insight into our understanding of SEC3 in the context of its participation in plant defense, which is a role that has not been identified in previous studies.

## Materials and methods

### Plant materials and growth conditions

Rice (*Oryza sativa* L. *japonica* var. Kitaake) and *N. benthamiana* plants were used in this study. All rice plants used in this study were grown in paddy fields of the Institute of Crop Science in Beijing, paddy fields of the Institute of Crop Science in Sanya (China), or a greenhouse at 25–28 °C with 16 h of light and 8 h of darkness every day.

### Sequence analysis and prediction of protein structure

Sequence analysis was performed using SMART (simple modular architecture research tool) (http://smart.embl-heidelberg.de/). The protein sequences used for the phylogenetic analysis and sequence alignment were obtained from the NCBI (http://www.ncbi.nlm.nih.gov/pmc/) and assessed using BLASTp software (http://blast.ncbi.nlm.nih.gov/Blast.cgi). Multiple sequence alignment was conducted with the DNAMAN software package (Lynnon BioSoft, Canada).

### Gene expression analysis

RNA extraction and quantitative reverse transcription–PCRs (qRT–PCRs) were performed as previously described (Ma *et al.*, 2016). mRNA was prepared using RNeasy plant mini kits (Qiagen, http://www.qiagen.com/). A 1 µg aliquot of mRNA was reverse-transcribed to cDNA using a QuantiTect reverse transcription kit (Qiagen, German). All qRT–PCR assays were carried out using the SYBR Premix Ex TaqKit (TaKaRa, Japan) with a 7900HT Real-time PCR System (Applied Biosystems, USA). Expression levels were calculated in three technical replicates consisting of two biological replicates each, with the rice *ubiquitin* gene serving as an internal control. The primers used in this study are listed in Supplementary Table S1 at *JXB* online.

For the β-glucuronidase (GUS) assay, 2.5 kb of the *OsSEC3A* promoter sequence was amplified using OsSEC3A-Pro-F/R, and the resulting construct was transformed into cv. Kitaake by *Agrobacterium tumefacien*s. Histochemical GUS staining was performed as previously described (Jefferson, 1987).

### Subcellular localization of OsSEC3A in rice roots and *N. benthamiana*

To obtain green fluorescent protein (GFP) transgenic plants, the OsSEC3A cDNA fragment was amplified using 1305OsSEC3A-GFP-F/R and cloned into the pCAMBIA1305-GFP vector (produced by insertion of the GFP backbone of pAN580 into the pCAMBIA1305 vector at the *Sac*I–*Sal*I sites) to generate the *pCAMBIA1305-D35S-OsSEC3A-GFP-NOS* vector. This construct was introduced into wild-type and *Ossec3a* mutant plants to generate transgenic plants. For brefeldin A (BFA) treatment, leaf epidermal cells of *N. benthamiana* were observed following incubation in 100 µg ml^–1^ BFA for periods of 20 min and 40 min. For transient expression analysis in *N. benthamiana*, the binary vector pCAMBIA1305-OsSEC3A-GFP (described above) was introduced into *Agrobacterium* strain EHA105, which was used to infiltrate *N. benthamiana* leaves as described previously; *N. benthamiana* protoplasts were isolated using the same method used for Arabidopsis (Ren *et al.*, 2014). Confocal images were acquired using a Leica TCS SP5 laser scanning confocal microscope.

### Transient expression analysis of rice protoplasts

Wild-type OsSEC3A cDNA fragments were amplified with primer pair OsSEC3A-GFP-F/R (Supplementary Table S1) and inserted into pAN transient expression vectors (http://www.bio.utk.edu/cellbiol/markers/vectors.htm) to produce OsSEC3A–GFP fusion proteins. The recombinant plasmid was co-transformed into rice protoplasts with plasma membrane markers. Transient expression using rice protoplasts from rice leaf sheaths was achieved as described previously (Shen *et al.*, 2014). Confocal images were acquired at 12 h after transformation using a Leica TCS SP5 system. For each experiment, >20 individual cells were imaged. For GFP, the excitation maximum was 488 nm, while the emission maximum was 530 nm. For monomeric red fluorescent protein (mRFP), the excitation maximum was 587 nm, while the emission maximum was 610 nm. Sequential imaging of both channels was employed. A single confocal slice was presented.

### Vector construction for the CRISPR/Cas9 system

The single guide RNA (sgRNA) design method for the CRISPR/Cas9 system in plants was described previously (Lei *et al.*, 2014). Here, a web application tool, CRISPR-P (http://cbi.hzau.edu.cn/crispr), was used to select sgRNAs targeting the third and 10th exon of the *OsSEC3A* gene. Plasmid construction was conducted as previously described. The Cas9 destination vector was driven by the maize ubiquitin promoter for expression in rice, and sgRNA expression was driven by the pol III type promoter of U3 sgRNA. Gateway recombination was used to mobilize sgRNA, after which the binary T-DNA vectors for co-expression of Cas9 and sgRNA were transformed into rice calli.

### Measurement of salicylic acid

Samples of seedling and leaf sheath tissue weighing ~0.3 g (FW) were prepared. Extraction and quantification of salicylic acid (SA) was performed as described previously, with minor modifications (Chen *et al.*, 1995).

### Pathogen infection assay

*Magnaportje oryzae* isolate HLJ07-45-1-2 (collected from Heilongjiang Province, China) was evaluated for virulence in Kitaake and the *Ossec3a* mutants. Four-week-old rice seedlings were spray-inoculated following a previously described procedure (Li *et al.*, 1998). The number and size of lesions in ~35 inoculated seedlings from each line were evaluated 7 days post-inoculation (dpi). Some of the seedlings were sampled at 0, 1, 2, 3, 4, and 5 dpi for qPCR. To quantify the disease symptoms, the disease severity, which reflects the percentage of the leaf area exhibiting necrosis/spots, was analyzed. Using ImageJ software, we quantified the disease severity (1–100%) of the first, second, third, and fourth fully expanded leaves for lines #5, #7, and #11. For the visualization of dead cells, the leaves of plants at the tillering stage were observed by trypan blue staining as previously described (Tang *et al.*, 2011). Hydrogen peroxide (H_2_O_2_) accumulation was detected by 3,3-diaminobenzidine (DAB) staining. H_2_O_2_ content was measured with a Hydrogen Peroxide Assay Kit (Beyotime, S0038) according to the manufacturer’s protocol.

### Assays for protein–protein interactions

For the yeast two-hybrid (Y2H) assay, the coding regions of *OsSEC3A* and various domain deletion variants of *OsSEC3A* were cloned into Y2H bait vector pGBKT7 (Clontech). The coding regions of other exocyst subunit genes were cloned into prey construct pGADT7 (Clontech) (see Supplementary Table S1 for primer sequences). Bait and prey constructs were co-transformed into *Saccharomyces cerevisiae* strain AH109. Positive clones grown on SD/-Leu/-Trp plates at 28 °C for 3 d were incubated in liquid media, after which a 10 μl drop of the culture dilution (OD_600_=0.5 with sterile water) was plated on SD/-Ade/-His/-Leu/-Trp plates to test the interaction between bait and prey.

For the *in vitro* pull-down assays, the coding regions of OsSEC3A, OsSEC3A^C^ (amino acids 310–888), OsSNAP32, OsExo70A1, and OsExo70B1 were inserted into pMAL-c2X and pGEX4T-1 vectors to generate maltose-binding protein (MBP)–OsSEC3A, MBP–OsSEC3^C^ (amino acids 310–888), glutathione *S*-trasferase (GST)–OsSNAP32, GST–OsExo70A1, and GST–OsExo70B1 plasmids, respectively (see Supplementary Table S1 for primer sets). Amylose resin (for MBP purification; New England Biolabs) and GST-binding resin (for GST purification; Merck) were used to purify proteins, including fusions and empty tags. The pull-down analyses were performed as previously described (Zhou *et al.*, 2013). Products were detected with an enhanced chemiluminescence reagent (GE Healthcare).

For the luciferase (LUC) reporter assay, the OsSEC3A__C_, full-length OsSNAP32, OsSEC5, OsSEC6, and OsSEC15b^N^ sequences were PCR amplified and inserted into *35S*:*NLuc* or *35S*:*CLuc* plasmids to construct LUC vectors as described previously (Chen *et al.*, 2008). These constructs were transiently introduced into *N. benthamiana* by infiltration with *A. tumefaciens* strain GV3101. After 3 d, *Agrobacterium*-infiltrated leaves were placed into a 96-well microtiter plate with a hole punch and kept in the dark for 5 min to allow detection of luminescence. Relative LUC activity was measured with the Dual-Luciferase Reporter Assay System (Promega).

### Lipid binding assay

The coding sequence (CDS) of OsSEC3A and OsSEC3A^N^ (amino acids 1–450) were amplified using primers (see Supplementary Table S1) and inserted into vector pMAL-c2X tagged with MBP using an infusion cloning kit (Clontech). Protein expression was induced with 200 µM isopropyl-β-d-thiogalactopyranoside at 16 °C overnight. Amylose affinity chromatography (New England Biolabs) was used to purify MBP-tagged proteins. The PIP strips/arrays (P-6001/ 6100; Echelon Biosciences) were blocked with 1% non-fat dry milk in phosphate-buffered saline (PBS) for 1 h and incubated in 10 μg ml^–1^ MBP-tagged recombinant protein in blocking buffer for 1 h at room temperature with gentle agitation. The blots were washed three times with PBS plus 0.1% Tween-20 (pH 7.4) and soaked in this buffer supplemented with 1:1000 murine horseradish peroxidase-conjugated anti-MBP antibodies (New England Biolabs) at room temperature for 1 h. After thorough washing, the signals were detected following the ECL Plus immunoblot method. As a negative control, we also incubated the membrane with the MBP tag only, and proceeded with the same experimental protocol.

## Results

### *SEC3A* is an active ortholog in rice

**Fig. 1.**
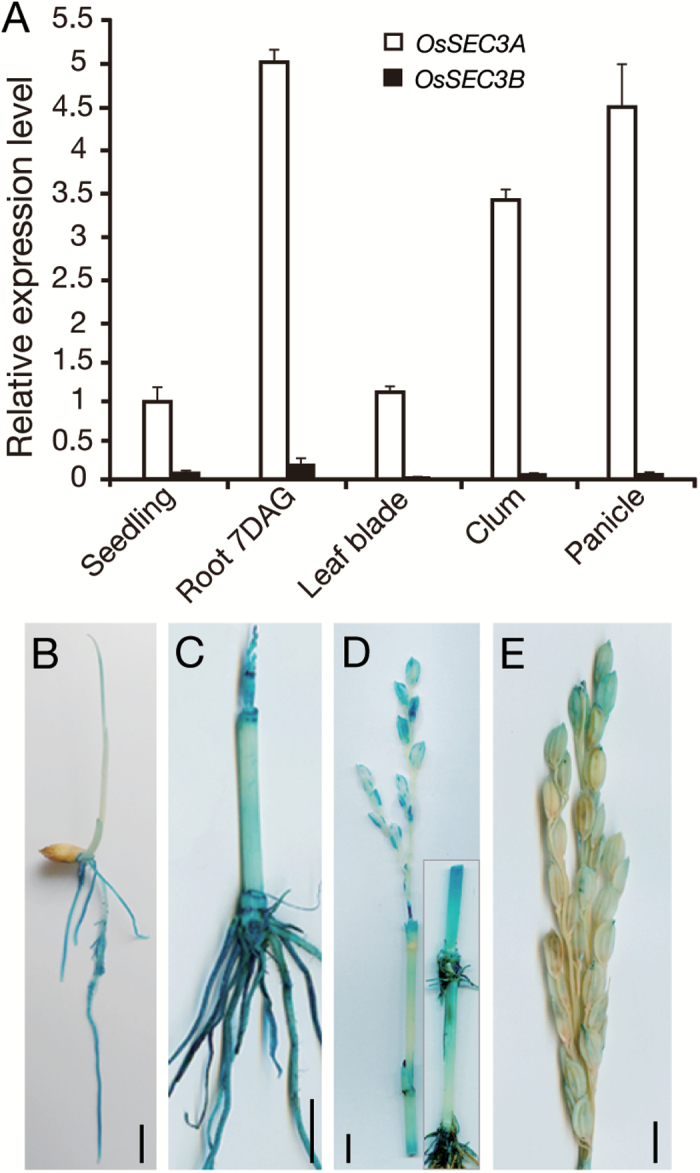
Expression analysis of OsSEC3A. (A) qRT–PCR expression patterns of OsSEC3A and OsSEC3B in various organs. Values are the means ±SE of three independent experiments. (B–E) Histochemical staining of young seedlings 4 d after germination (B), at 4 weeks (C), 1 week before heading (D), and at the heading stage (E). The image inserted in (D) is another part of the same transgenic plant. Scale bars=1 cm in (B)–(E).

Rice exocyst subunit *SEC3* has two orthologous genes, *OsSEC3A* (LOC_Os03g42750) and *OsSEC3B* (LOC_Os11g17600), which share 64% sequence identity at the protein level (Cvrčková *et al.*, 2012). The expression patterns of OsSEC3A and OsSEC3B were determined by qRT–PCR. OsSEC3A was expressed in various organs, including the roots, culms, panicles, and leaves, with the highest expression in the seedling roots, whereas almost no *OsSEC3B* transcript was detected (Fig. 1A). GUS expression driven by the *OsSEC3A* promoter confirmed the constitutive expression pattern detected by qRT–PCR and further indicated that GUS staining was higher in young roots (Fig. 1B, C), shoot apices (Fig. 1C), the basal parts of internodes (divisional and elongating zones) originating from the intercalary meristem (Fig. 1D), and young glumes (Fig. 1D, E). The broadly high expression of *OsSEC3A* in organs examined in our analysis indicated that OsSEC3A is the major SEC3 paralog in rice. Next, we focused our work mainly on rice exocyst subunit OsSEC3A.

### OsSEC3A is a member of the exocyst complex

**Fig. 2.**
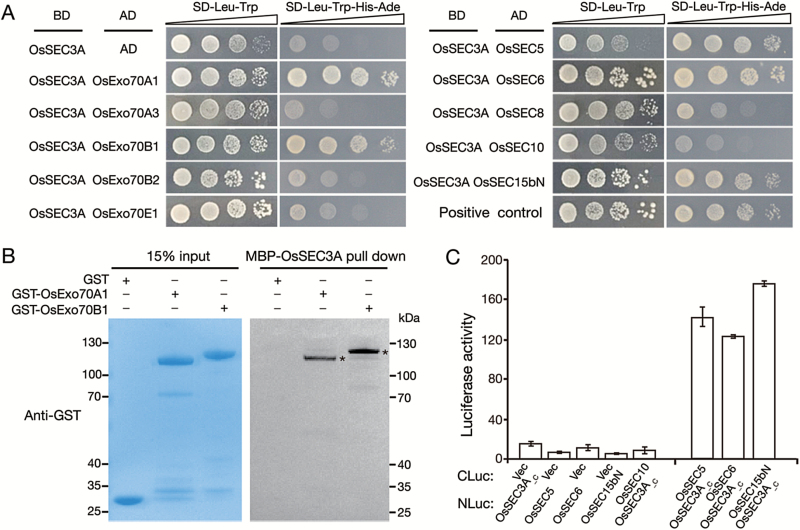
Interaction of OsSEC3A with other exocyst subunits. (A) Y2H assay in *S. cerevisiae*. Full-length OsSEC3A was ligated into pGBKT7 to generate the OsSEC3A-BD plasmid. Several exocyst constructs were fused with the activation doamin (AD), displaying no auto-activation. (B) *In vitro* pull-down assay of recombinant GST–OsExo70A1, GST–OsExo70B1, and GST using resins containing MBP–OsSEC3A. Asterisks indicate GST–OsExo70A1 and GST–OsExo70B1. (C) Quantification of luciferase activity in *N. benthamiana* leaves expressing: OsSEC3A__C_ (amino acids 521–888)-NLuc and CLuc-OsSEC5; OsSEC3A__C_-NLuc and CLuc-OsSEC10; OsSEC3A__C_-NLuc and CLuc-OsSEC6; or OsSEC3A__C_-NLuc and CLuc-OsSEC15BN. The data shown are representative of three independent experiments.

To gain further insight into whether exocyst subunit OsSEC3A functions as a component of the exocyst complex, we performed Y2H assays with a group of subunits that do not display auto-activation (Supplementary Fig. S1). OsSEC3A interacted with OsExo70A1 and OsExo70B1. There was no interaction between OsSEC3A and OsExo70A3, OsExo70B2, or OsExo70E1 (Fig. 2A). In addition, our *in vitro* pull-down assay verified that MBP–OsSEC3A pulled down GST–OsExo70A1 and GST–OsExo70B1 fusion proteins, but not GST itself (Fig. 2B). For other exocyst subunits, OsSEC3A interacted with OsSEC5, OsSEC6, and OsSEC15bN in Y2H assays (Fig. 2A). We further used a split LUC complementation assay based on firefly LUC (Chen *et al.*, 2008) to confirm this interaction. Consistently, enhanced LUC activity was detected in *N. benthamiana* leaves (Fig. 2C). Taken together, these findings show that OsSEC3A might have the ability to participate in exocyst complexes.

### Mutation of OsSEC3A causes dwarfism and lesion spots

**Fig. 3.**
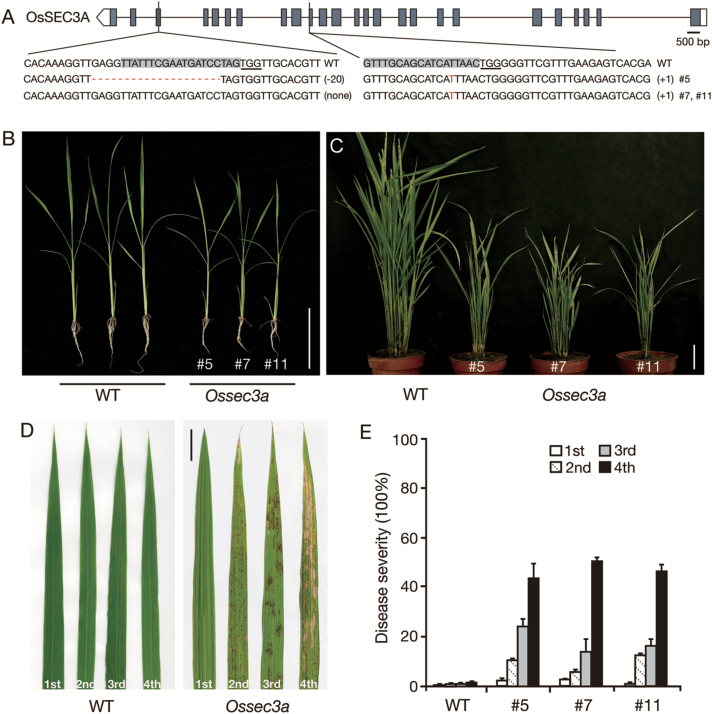
Phenotypes of field-grown wild-type (WT) and *Ossec3a* mutant plants. (A) Targeted mutagenesis of the *OsSEC3A* gene. The positions of target sites are shown on the gene structure. The protospacer adjacent motif (PAM) sequence is underlined, while the target sequence is shaded in gray. The sequencing results of mutant alleles are aligned to the reference genome sequence. The indels are shown in red letters or dashes. The net change in length is noted to the right of each sequence (+, insertion; –, deletion). (B and C) Phenotype of WT and *Ossec3a* mutant plants at the seedling (B) and heading stage (C). (D) Phenotype of the first, second, third, and fourth fully expanded leaves from the tip to the base of the main tiller of plants at the heading stage. (E) Quantification of disease symptoms for leaves of different ages. Disease severity reflects the percentage of the leaf area exhibiting necrosis/spots. Scale bars=10 cm in (B, C) and 5 cm in (D).

To decipher the functional role of OsSEC3A in plant development, CRISPR/Cas9 technology, the application of which has been demonstrated to achieve efficient targeted mutagenesis in transgenic rice (Miao *et al.*, 2013), was used to disrupt specifically the *OsSEC3A* gene. To enhance mutation efficiency, we designed sgRNAs targeting the third and 10th exon of the *OsSEC3A* gene and transformed them into *O. japonica* var. Kitaake simultaneously (see the Materials and methods). Twenty independent transgenic lines for the sgRNA construct were obtained, after which DNA sequencing analysis of these lines was conducted using gene-specific primers. The sequencing analysis showed either loss of peak/gain of peak or overlapping peaks around the target site in the sequencing chromatograms of five lines: #2, #5, #7, #11, and #18 (Fig. 3A; Supplementary Fig. S2), which confirmed mutations in the *OsSEC3A* gene. In addition, qRT–PCR analyses showed severely reduced abundance of *OsSEC3A* as expected (Supplementary Fig. S3). The occurrence of the detected mutation upstream of the protospacer adjacent motif (PAM) indicated that the CRISPR/Cas9 system cleaved the target sequence.

Detailed phenotype analysis showed that each independent transgenic line (lines #5, #7, and #11) exhibited similarly altered growth in comparison with that of wild-type plants (Fig. 3). The progeny of transgenic line #5 were selected for the following studies and designated *Ossec3a*. In the seedling stage, *Ossec3a* plants were smaller than wild-type seedlings (Fig. 3B). The length of the main roots of *Ossec3a* plants was reduced in comparison with those of wild-type plants (Supplementary Fig. S4A). Lesions appeared on *Ossec3a* leaf blades and leaf sheaths from the tillering stage to the ripening stage (Fig 3C; Supplementary Fig. S4B). The disease severity of the lesions formed on *Ossec3a* mutants appeared to follow a developmental pattern and was correlated with the age of the affected leaf blades. No spots were present on newly emerging flag leaves, whereas the second leaf blades from the top of each main tiller had larger and more numerous spots, while the third and fourth leaves showed some withering (Fig 3C–E). However, the corresponding leaf blades of wild-type plants showed no spots in all developmental stages (Fig 3C–E). When the last emerging flag leaf was filled with spots, the *Ossec3a* spots began to senesce far earlier than those of the wild-type plants (Fig 3C, D). In addition to an obvious leaf blade phenotype, several agricultural traits, including plant height, panicle length, tiller number, thousand-grain weight, and spikelet fertility, were decreased/impaired in the *Ossec3a* mutants in comparison with the wild-type plants (Supplementary Fig. S5).

The lesion phenotype of *Ossec3a* mutant plants was fully recovered when the full-length *OsSEC3A* cDNA was introduced into *Ossec3a* mutant line #5 under the control of the 35S promoter (Supplementary Fig. S4C). This result indicates that the lesion phenotype was due to a loss of function of *OsSEC3A*. The results of the experiments with *OsSEC3A* RNAi (T_3_ homozygous) showed a lesion phenotype similar to that of the *Ossec3a* mutant plants (Supplementary Fig. S4D). qRT–PCR analyses showed severely reduced abundance of *OsSEC3A* (~5% of the normal transcript level), whereas *OsSEC3B* and *OsSEC5* transcript levels were not affected (Supplementary Fig. S4E), demonstrating that the RNAi vector was specific for *OsSEC3A*.

### *Ossec3a* displays induced cell death

**Fig. 4.**
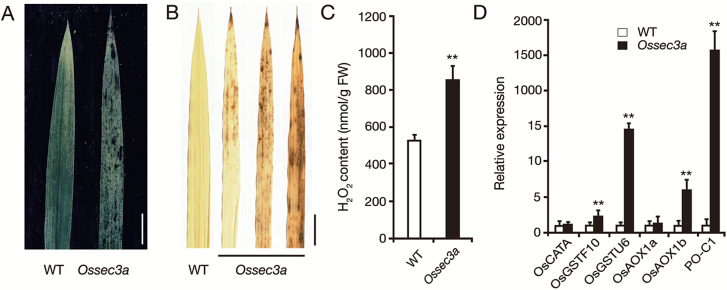
*Ossec3a* displays induced cell death. (A) Staining of the leaf blades of wild-type (WT) and *Ossec3a* mutant plants with trypan blue. (B) DAB staining in WT and *Ossec3a* leaf blades. Scale bars=5 cm in (A, B). (C) Quantitative measurements of H_2_O_2_ in the leaves of WT and *Ossec3a* mutant plants. (D) qRT–PCR analysis of antioxidant enzyme genes in WT and *Ossec3a* plants. Significance was assessed using the Student’s *t*-test (**P<0.01). Error bars indicate the SD.

The appearance of lesion spots in the *Ossec3a* mutant resembled that of LMMs, which usually exhibit immunity-mediated cell death. Trypan blue staining revealed that the lesions of *Ossec3a* mutants contained large areas of dead cells, whereas no blue staining was observed in wild-type leaves (Fig. 4A). To confirm this result, we analyzed production of H_2_O_2_, another marker of cell death. We observed intense staining in *Ossec3a* leaves following treatment with DAB, whereas no DAB signal was found in wild-type leaves (Fig. 4B). Consistently, the H_2_O_2_ content in *Ossec3a* leaves was 1.6-fold (852.3 ± 83.5 for the wild type; 529.4 ± 28.7 for *Ossec3*) higher than that of wild-type leaves (Fig. 4C). In addition, the expression level of hydrogen peroxidase PO-C1 (Wally *et al.*, 2009) was significantly increased in *Ossec3a* mutants. Transcript levels of OsAOX1b (LI *et al.*, 2013), OsGSTU6, and OsGSTF10 (Jardim-Messeder *et al.*, 2015), which have been shown to be induced by H_2_O_2_, were also up-regulated in *Ossec3a* mutants, consistent with H_2_O_2_ accumulation (Fig. 4D). These results demonstrate that cell death is induced in *Ossec3a* mutants in a manner similar to that observed in LMMs.

### *Ossec3a* mutants display an enhanced defense response

**Fig. 5.**
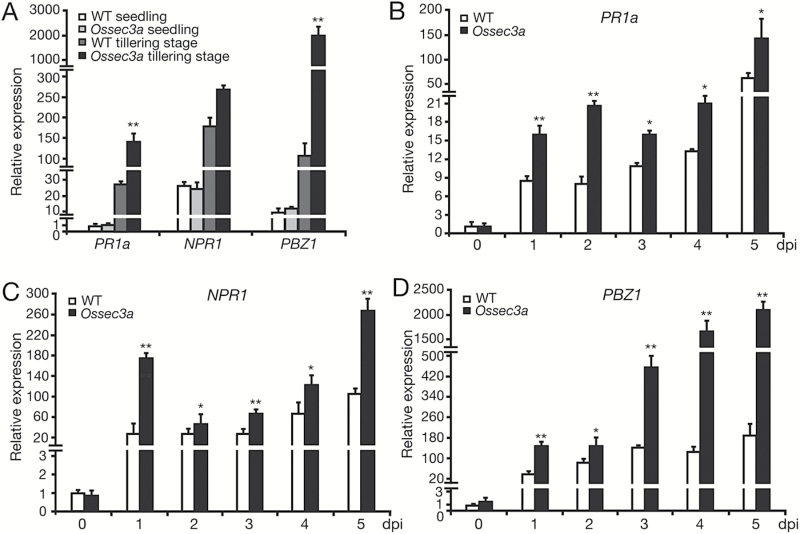
Expression of pathogenesis-related genes in wild-type (WT) and *Ossec3a* mutant plants. (A) qRT–PCR of pathogenesis-related genes in WT and *Ossec3a* plants. Samples were detached from leaf blades at the seedling stage and tillering stage when lesions appeared. (B–D) qRT–PCR of pathogenesis-related genes *PR1a* (B), *NPR1* (C), and *PBZ1* (D) using 4-week-old WT and *Ossec3a* plants at various time points from 0 to 5 dpi (days post-inoculation) with *Magnaporthe oryzae*. Values are presented as the mean ±SE of three independent experiments. Significant differences were determined with the Student’s *t*-test (*0.01<*P*<0.05; ** *P*<0.01).

It has been reported that defense responses are activated during lesion formation in rice LMMs (Manosalva *et al.*, 2011). To address this possibility in *Ossec3a* mutant plants, we measured the transcript levels of pathogenesis-related (PR) genes, including *PR1a*, *NPR1*, and *PBZ1.* At the seedling stage, when lesions had not developed, transcript levels of PR genes were very low and no difference was detected between *Ossec3a* and wild-type leaf blades. However, 3 d after the appearance of lesions of the tillering stage, *PR1a* and *PBZ1* were up-regulated significantly in the leaf blades of *Ossec3a* mutants in comparison with corresponding wild-type leaf blades (Fig. 5A). We also examined PR gene expression at various time points from 0 to 5 dpi with *M. oryzae* in *Ossec3a* mutants and wild-type plant seedlings. After spray-inoculation, the relative transcript levels of PR genes were increased and found to be much higher in *Ossec3a* mutants in comparison with those of wild-type plants; in particular, transcript abundance of *PBZ1* was increased markedly in *Ossec3a* mutants at 4 and 5 dpi in comparison with that of wild-type plants (Fig. 5B–D).

**Fig. 6.**
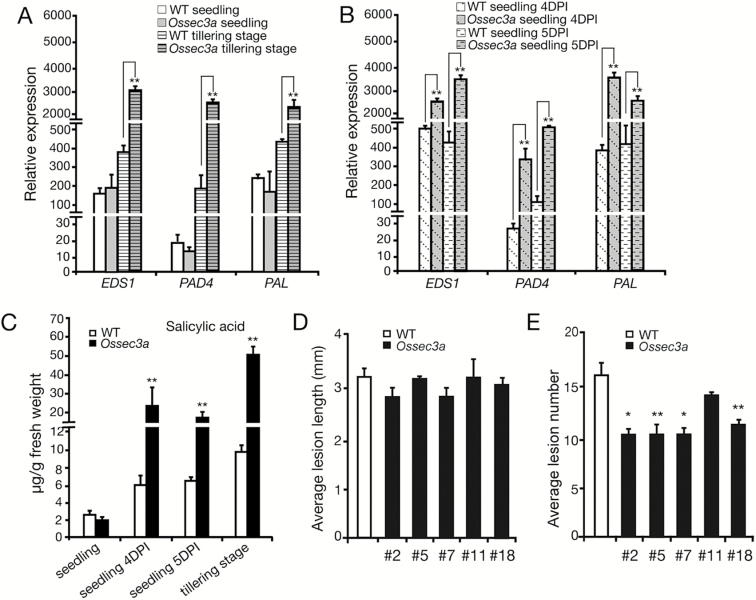
Accumulation of salicylic acid (SA) and enhanced disease resistance to *Magnaporthe oryzae* in the *Ossec3a* mutant plants. (A and B) qRT–PCR of SA synthesis-related genes *EDS1*, *PAD4*, and *PAL* in *Ossec3a* seedlings or tillering plants without (A) and with (B) *M. oryzae* inoculation. (C) Levels of free SA in *Ossec3a* and wild-type (WT) plants with and without *M. oryzae* inoculation. (D and E) Evaluation of resistance to *M. oryzae* in WT and *Ossec3a* mutant plants. The average lesion length (D) and number (E) in WT and *Ossec3a* mutant (line #2, #5, #7, #11, and #18) leaves at 7 dpi are shown. The bars represent the SE of three replicates. Significance was determined with the Student’s *t*-test (*0.01<*P*<0.05; **P<0.01).

As a molecular marker for defense responses, SA accumulates in leaves and activates PR genes (Durrant and Dong, 2004). To investigate this role of SA, we examined transcript levels of SA synthesis-related genes *EDS1* (enhanced disease susceptibility 1), *PAD4* (phytoalexin deficient 4), and *PAL* (phenylalanine ammonia-lyase) (Du *et al.*, 2009). At the seedling stage, no differences between *Ossec3a* and wild-type leaves were detected. However, after inoculation with *M. oryzae*, SA synthesis-related genes were up-regulated significantly in *Ossec3a* seedlings in comparison with those of wild-type seedlings (Fig. 6A, B). The same up-regulation of these genes was also observed in the *Ossec3a* mutants at the tillering stage (Fig. 6A). We also measured the abundance of free SA using HPLC. As expected, no difference was observed in the abundance of SA in *Ossec3a* and wild-type seedlings. However, at the tillering stage and after inoculation with *M. oryzae*, *Ossec3a* seedlings had ~3.0- to 5.5-fold higher free SA in comparison with wild-type seedlings (Fig. 6C). The observed up-regulation of defense response-related genes and accumulation of SA in response to pathogen inoculation indicated that plant defense responses were enhanced in *Ossec3a* mutant plants.

Another molecular marker commonly known to participate in plant defense responses is jasmonic acid (JA) (Glazebrook, 2001). Therefore, we explored the expression patterns of genes related to JA biosynthesis and signaling. The results were consistent with those of our experiments assessing expression of SA-related genes. Significant up-regulation of JA biosynthesis gene *OsLOX2.2* and JA signaling genes *OsJAZ1* and *OsJAZ2* was observed in *Ossec3a* leaves at the tillering stage, as well as in *Ossec3a* seedlings with inoculation of *M. oryzae* (Supplementary Fig. S6). No changes were detected between wild-type and *Ossec3a* seedlings without inoculation (Supplementary Fig. S6). These results indicated activation of JA synthesis and the JA signaling pathway, which is consistent with the idea that *Ossec3a* mutants have an enhanced defense response.

To test whether *Ossec3a* plants show actual resistance to pathogens, we spray-inoculated *Ossec3a* and wild-type plants with an *M. oryzae* isolate at the seedling stage. One week after inoculation, *Ossec3a* mutants and wild-type plants showed typical lesions of similar length (Fig. 6D). However, the leaves of *Ossec3a* mutants had fewer lesions than did the wild-type seedlings (Fig. 6E), suggesting that *Ossec3a* mutant plants exhibited enhanced disease resistance against *M. oryzae.*

### OsSEC3A localizes with the plasma membrane

**Fig. 7.**
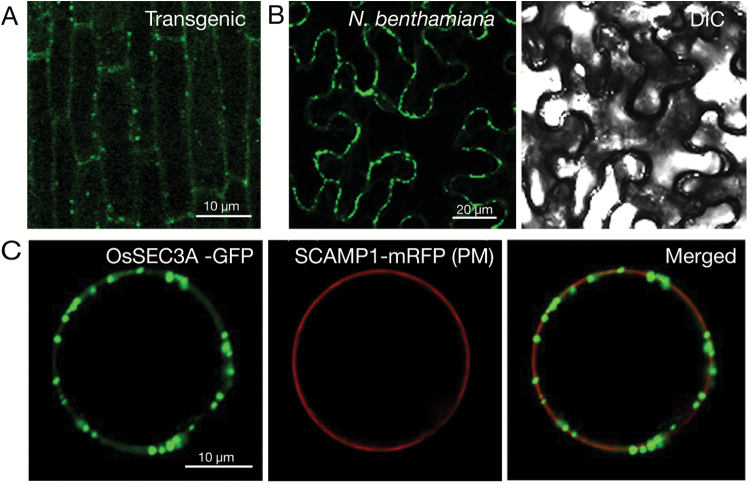
Subcellular localization of OsSEC3A–GFP. (A) Confocal microscopy of OsSEC3A–GFP in the root epidermal cells of *OsSEC3A* transgenic seedlings. (B) Confocal microscopy of OsSEC3A–GFP in the leaf epidermal cells of *N. benthamiana*. (C) Confocal micrograph of the distributions of OsSEC3A–GFP and the indicated markers (SCAMP1–mRFP; plasma membrane) 12 h after transformation.

To elucidate the subcellular localization of OsSEC3A, a 35S:OsSEC3A:GFP construct, with the C-terminus of OsSEC3A connected with the N-terminus of GFP, was expressed under the control of the 35S promoter and transformed into rice calluses. Under a fluorescence microscope, we observed strong GFP signals in punctate structures in the periphery of root epidermal cells in OsSEC3A transgenic seedlings (Fig. 7A). A similar subcellular localization pattern was observed in leaf epidermal cells of *N. benthamiana* (Fig. 7B). To confirm further that these punctate structures accumulated in the plasma membrane, we transiently transformed OsSEC3A–GFP into rice protoplasts with a plasma membrane marker (SCAMP–mRFP; Lam *et al.*, 2007). The punctate OsSEC3A–GFP signals were localized within the plasma membrane signal (Fig. 7C). These results indicated that OsSEC3A exhibited a punctate distribution and was most probably localized within, or linked to, the plasma membrane.

A previous study reported plasma membrane localization of OsSNAP32, a SNARE protein that mediates membrane fusion, in rice (Bao *et al.*, 2008). In agreement with these findings, our results provide solid evidence for interaction between OsSEC3A and OsSNAP32 (see below), in accordance with the idea that OsSEC3A localizes with the plasma membrane.

### OsSEC3A binds to phosphatidylinositols through its N-terminus

**Fig. 8.**
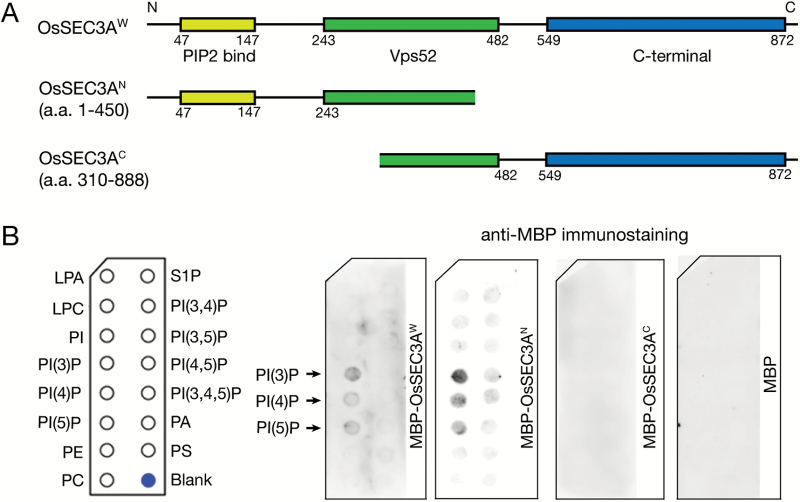
Lipid binding assays using the full-length OsSEC3A protein and different domains. (A) Schematic structure of the full-length OsSEC3A protein, including three predicted domains and two segmentations, used for purifying recombinant proteins. (B) Purified recombinant proteins with the MBP tag were used to probe PIP strips. Interactions are indicated by arrows. Lipid binding was assayed with three biological replicates. LPA, lysophosphatidic acid; LPC, lysophosphocholine; PI, phosphatidylinositol; PE, phosphatidylethanolamine; PC, phosphatidylcholine; S1P, sphingosine 1-phosphate; PA, phosphatidic acid; PS, phosphatidylserine.

In yeast and humans, the Sec3 subunit anchors the exocyst complex to the plasma membrane via interaction with phosphatidylinositol (He *et al.*, 2007; Liu *et al.*, 2007; Zhang *et al.*, 2008). The pleckstrin homology (PH) domain in the N-terminus of the Sec3 subunit plays a critical role in binding to the plasma membrane (He *et al.*, 2007; Liu *et al.*, 2007). To test whether OsSEC3A works in a similar manner, we performed a bioinformatics analysis on the encoding gene. The results of this analysis showed that *OsSEC3A* encodes a protein composed of 888 amino acid residues, containing a PIP2-binding domain (amino acids 47–147, corresponding to the PH domain), a Vps52 domain (amino acids 243–482), and the C-terminal region (amino acids 549–872) (Fig. 8A). Amino acid sequence alignment analysis showed that the key amino acids responsible for membrane binding are highly conserved (Supplementary Fig. S7). This implies that the N-terminus of OsSEC3A should bind phospholipids as demonstrated in previous studies (Bloch *et al.*, 2016). Therefore, we implemented a lipid binding assay using purified recombinant MBP–OsSEC3A^W^ protein (whole protein), MBP–OsSEC3A^N^ (amino acids. 1–450, N-terminal half), and MBP–OsSEC3A^C^ (amino acids 310–888, C-terminal half). The results of this assay showed obvious binding of MBP–OsSEC3A^W^ and MBP–OsSEC3A^N^ to phosphatidylinositol, whereas MBP–OsSEC3A^C^ showed no binding signal (Fig. 8B). These results suggest that OsSEC3A, similarly to the SEC3 subunit in humans and yeast, has the ability to bind phospholipids through its N-terminus, which supports our observation that OsSEC3A localizes in or at the plasma membrane.

### OsSEC3A interacts with OsSNAP32 through its C-terminus

**Fig. 9.**
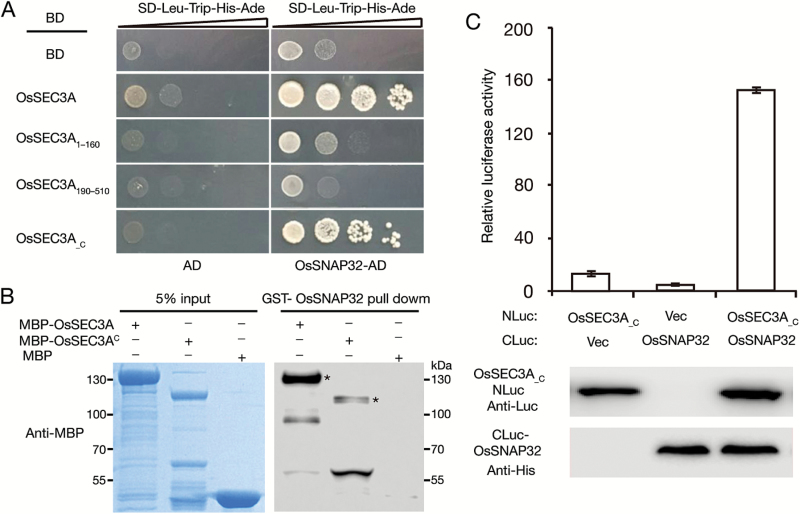
OsSEC3A physically interacts with OsSNAP32. (A) Y2H assay showing that full-length OsSEC3A and OsSEC3A__C_ (amino acids 521–888) interact with OsSNAP32, while two other predicted domains, OsSEC3A_1–160_ (including the PH domain) and OsSEC3A_190–510_ (including Vps52) failed to interact with OsSNAP32. (B) *In vitro* pull-down assay of recombinant MBP–OsSEC3A, MBP–OsSEC3A^C^ (amino acids 310–888, C-terminal half), and MBP using resins containing GST–OsSNAP32. Asterisks indicate MBP–OsSEC3A and MBP–OsSEC3A^C^ proteins. (C) Quantification of luciferase (LUC) activity in *N. benthamiana* leaves expressing OsSEC3A__C_-NLuc and CLuc-OsSNAP32. The western blot shows the expression levels of CLuc and NLuc fusion proteins. OsSEC3A__C_ derivatives were detected by anti-firefly LUC antibodies, whereas CLuc-OsSNAP32 was detected by anti-His antibodies. The staining shows equal loading of protein in each lane. Data were collected 3 d after infiltration. The data shown are representative of three independent experiments.

To explore how and why *OsSEC3A* participates in plant immunity and defense responses, we assessed whether OsSEC3A interacts with a group of known immunity- and defense-related protein factors, including SPL11, SPL28, OsVAMP721, OsSNAP32, OsPUB51, and ORPM1 (Zeng *et al.*, 2004, 2008; Qiao *et al.*, 2010). Our Y2H assays revealed interaction between OsSNAP32 and OsSEC3A (Fig. 9A; Supplementary Fig. S8), whereas no interaction was detected between OsSEC3A and any of the other tested proteins (Supplementary Fig. S8). OsSNAP32 is a SNAP25-type SNARE protein involved in resistance to rice blast (Luo *et al.*, 2016). Considering that SNAREs play important roles in plant responses to various biotic and abiotic stresses (Collins *et al.*, 2003; Kwon *et al.*, 2008), and *Ossec3a* plants exhibited enhanced defense response, including resistance to rice blast (Fig. 5; Supplementary Fig. S6), we boldly speculated that OsSEC3A might function in a SNARE complex-mediated pathway. Furthermore, our deletion analysis revealed that the C-terminus, OsSEC3A__C_ (amino acids 521–888), displayed a specific interaction with OsSNAP32 (Fig. 9A), whereas the other domains, OsSEC3A_1–160_ (including the PH domain) and OsSEC3A_190–510_ including Vps52), failed to interact with OsSNAP32 (Fig. 9A). These results indicate that the C-terminal domain of OsSEC3A was essential for mediating the interaction between OsSEC3A and OsSNAP32. We further performed *in vitro* pull-down assays and split LUC complementation assays to verify this conclusion. The results of the *in vitro* pull-down assays confirmed that OsSNAP32–GST pulled down MBP–OsSEC3A and MBP–OsSEC3A^C^ (amino acids 310–888) (Fig. 9B). In the split LUC complementation assay, we showed that LUC activity in *N. benthamiana* leaves that co-expressed *Pro35S:OsSEC3A*_*_C*_*-NLuc* and *Pro35S:CLuc-OsSNAP32* was ~15-fold greater than that of leaves expressing two negative vector controls (*Pro35S:OsSEC3A*_*_C*_*-NLuc* and *Pro35S:CLuc* or *Pro35S:NLuc* and *Pro35S:CLuc*-*OsSNAP32*) (Fig. 9C). These results confirmed the interaction between OsSNAP32 and OsSEC3A through the C-terminal region (OsSEC3A__C_; amino acids 521–888)_._

## Discussion

### OsSEC3A participates in plant defense responses

SEC3 is a subunit of the exocyst complex that recruits other subunits residing in the cytoplasm to the plasma membrane (Finger *et al.*, 1998; Wiederkehr *et al.*, 2003). Previous studies in maize and Arabidopsis reveal that mutations in SEC3 result in failure to achieve correct root hair elongation (Wen *et al.*, 2005) and defective pollen tube growth (Bloch *et al.*, 2016). These observations of compromised polarized growth in plants with mutated SEC3 indicate that SEC3 plays a role in polar exocytosis. The novelty of this study is that we show for the first time that SEC3 has a role in the plant defense response in rice. Although the *Ossec3a* mutant rice plants showed slower growth of the main roots in comparison with that of wild-type plants, no specific inhibition of root hair growth was observed (Fig. 3B). Moreover, defects in pollen tube growth were not observed because the homozygous *Ossec3a* mutant plants produced normal seeds. Taking these results into account, it is likely that OsSEC3A may not be required for polarized growth. Instead, the *Ossec3a* mutants in our study presented a phenotype resembling that of LMMs. Lesions appeared on *Ossec3a* leaf blades and leaf sheaths from the tillering stage to the ripening stage (Fig. 3; Supplementary Fig. S4), and cell death was induced in *Ossec3a* mutants in a manner similar to that observed in LMMs (Fig. 4). In addition, *Ossec3a* mutants also showed signs of enhanced immunity, including enhanced SA content, up-regulation of defense response-related genes, and enhanced disease resistance against *M. oryzae* (Figs 5, 6).

 Recently, ~15 LMM mutants showing enhanced immunity and spontaneous cell death were identified in rice (Zhu *et al.*, 2016). The well-studied proteins responsible for the LMM phenotype are E3 ubiquitin ligase SPL11 and AAA ATPase LRD-6. The former protein negatively regulates plant defense by associating with SPIN6 and OsRac1, and the latter probably inhibits immunity by regulating multivesicular body-mediated vesicular trafficking (Zeng *et al.*, 2004; Zhu *et al.*, 2016). In this study, *Ossec3a* mutants showed spontaneous lesions and enhanced resistance to disease, which are the most typical phenotypes of LMMs. This finding implies a possible interaction between OsSEC3A and the molecular pathway of LMM immunity.

### Localization of OsSEC3A and interaction with other exocyst subunits

In Arabidopsis, SEC3A, as well as other exocyst subunits such as SEC6, SEC8, and EXO70A1, have been shown to localize at the plasma membrane with a punctate distribution (Žárský *et al.*, 2013; Zhang *et al.*, 2013). Our results showed almost the same localization pattern for OsSEC3A (Fig. 7). The distribution of SEC3A and other exocyst subunits in Arabidopsis can occur in a manner dependent on (Zhang *et al.*, 2013) or independent of (Fendrych *et al.*, 2013) secretory vesicles. In this study, using the inhibitor BFA, which causes aggregation of the *trans*-Golgi network and inhibits exocytosis (Wang *et al.*, 2016), we showed that, upon BFA treatment, the punctate-localized OsSEC3A–GFP signal maintained the same pattern as that observed in untreated plants, which differed from that of the positive control Man1–mRFP (*cis*-Golgi marker), which was completely redirected to the endoplasmic reticulum (Supplementary Fig. S9). This finding suggests that localization of OsSEC3A is independent of the presence of secretory vesicles, in agreement with a previous study of Arabidopsis (Fendrych *et al.*, 2013).

Previous studies demonstrate that SEC3A functions as a subunit of the exocyst complex (Zhang *et al.*, 2013). Membership of a protein complex such as the exocyst is usually determined by assessing protein–protein interactions with the other subunits in the complex. In Arabidopsis, SEC3A interacts with EXO70A1 and SEC5A in the exocyst complex (Zhang *et al.*, 2013). In rice, however, we showed that OsSEC3A interacts with EXO70B1, SEC5A, SEC6, and SEC15B (Fig. 2). Notably, Arabidopsis SEC3A does not interact with SEC6 or SEC15B (Zhang *et al.*, 2013). Our results showed that OsSEC3A also failed to interact with EXO70A1 (Fig. 2). We were unable to locate any further information about the subunits that interact with SEC3A in other plant species. Thus, our results provide some initial insight into exocyst assembly in plants. The additional subunits that were observed to interact with OsSEC3A, SEC6, and SEC15 (besides EXO70 and SEC5 in Arabidopsis) may reflect the existence of exocyst subcomplexes in rice.

### Possible links between OsSEC3A and plant defense

In plants, the function of the exocyst is linked to normal development and defense response (Žárský *et al.*, 2013; Fujisaki *et al.*, 2015; Zhao *et al.*, 2015). Among the subunits that comprise the exocyst complex, EXO70B1 is a unique subunit of the exocyst complex, whose mutant shows the lesion-mimic phenotype and enhanced disease resistance (Stegmann *et al.*, 2013; Zhao *et al.*, 2015). In our study, we found that mutation of *OsSEC3A* caused spontaneous leaf lesions resembling those caused by hypersensitive response (HR) in the absence of pathogen infection. This phenotype of *Ossec3a* mutants is very similar to that of *exo70B1* mutants. It is interesting that mutations of *SEC3A* in other plants species, such as maize and Arabidopsis, do not produce a similar phenotype (Wen *et al.*, 2005; Zhang *et al.*, 2013). To explore how and why *OsSEC3A* is involved in plant immunity and defense responses, we examined the possibility that OsSEC3A interacts with a group of known immunity- and defense-related protein factors. We discovered that OsSEC3A interacts directly with OsSNAP32 (Fig. 9), a SNAP25-type SNARE protein that influences the resistance of rice to disease (Luo *et al.*, 2016). This finding provides a molecular link between OsSEC3A and plant immunity and defense responses. In Arabidopsis, AtSNAP33, an OsSNAP32 homolog, is involved in immune responses by playing a role in the formation of papilla below infection sites (Kwon *et al.*, 2008; Yun *et al.*, 2013). Moreover, *AtSNAP33* mutants show a lesion-mimic phenotype. According to these findings, beyond our initial report of the involvement of SEC3A in plant immunity, we hypothesized that SNARE complex-mediated signaling might be critical for this function.

Using lipid binding assays, we showed that OsSEC3A binds to PI(3)P; the N-terminus of OsSEC3A was essential for this binding (Fig. 8). Strikingly, this type of binding is different from that through which PI(4,5)P_2_ interacts with Sec3 in yeast (Zhang *et al.*, 2008). The latter type of lipid binding to PI(4,5)P_2_ has also been observed in Arabidopsis, although the sequence similarity of SEC3A in Arabidopsis and rice is high (Bloch *et al.*, 2016). It is notable that external PI(3)P is reported to mediate the entry of pathogen effectors into plant host cells (Kale *et al.*, 2010; Helliwell *et al.*, 2016). This may suggest another link between OsSEC3A and the defense response. In addition, the difference in phosphatidylinositol binding may reflect differences in the manner in which SEC3As participate in developmental processes; Arabidopsis SEC3A probably participates in pollen tube growth and embryo development, whereas OsSEC3A probably participates in plant defense.

## Supplementary data

Supplementary data are available at *JXB* online.

Fig. S1. Several exocyst subunits fused with the activation domain displayed no auto-activation in our experiments.

Fig. S2. Alignment of mutations in *OsSEC3A* from chromatograms to the reference genome sequence.

Fig. S3. qRT–PCR analysis of *OsSEC3A* in wild-type and *Ossec3a* plants (#5, #7, #11).

Fig. S4. Performance of whole plants (wild-type and transgenic lines) at different developmental stages.

Fig. S5. Agronomic traits (plant height, panicle length, tiller number, thousand-grain weight, and spikelet fertility) of wild-type and *Ossec3a* mutant plants.

Fig. S6. qRT–PCR of jasmonic acid synthesis-related genes (*OsLOX2.2* and *OsJAR1;2*) and signaling pathway genes (*OsJAZ1* and *OsJAZ2*) in wild-type and *Ossec3a* plants.

Fig. S7. Evolutionary analysis of OsSEC3A.

Fig. S8. Interaction of OsSEC3A with a group of known immunity- and defense-related protein factors.

Fig. S9. BFA treatment of leaf epidermal cells co-expressing OsSEC3A–GFP and Man1–mRFP.

Table S1. Sequences of DNA oligonucleotides used in this study

Supplementary Figures and Table

## Abbreviations

BFA: brefeldin A
DAB: 3,3′-diaminobenzidine
dpi: days post-inoculation
H_2_O_2_: hydrogen peroxide
LUC: luciferase
PH: pleckstrin homology
PI(3)P: phosphatidylinositol-3-phosphate
PI(4,5)P_2_: phosphatidylinositol 4,5-bisphosphate
PR: pathogenesis-related
SA: salicylic acid
SNAP: soluble *N*-ethylmaleimide-sensitive factor attachment protein
VAMP: vesicle-associated membrane protein
Y2H: yeast two-hybrid.
