# Corrigendum: Disruption of OsSEC3A increases the content of salicylic acid and induces plant defense responses in rice

**DOI:** 10.1093/jxb/ery003

**Published:** 2018-01-25

**Authors:** Jin Ma, Jun Chen, Min Wang, Yulong Ren, Shuai Wang, Cailin Lei, Zhijun Cheng, Sodmergen

**Affiliations:** 1 Key Laboratory of Ministry of Education for Cell Proliferation and Differentiation, College of Life Sciences, Peking University, Beijing, China; 2 National Key Facility for Crop Gene Resources and Genetic Improvement, Institute of Crop Science, Chinese Academy of Agricultural Sciences, Beijing, China

*Journal of Experimental Botany*, advance access: December 2017, doi: 10.1093/jxb/erx458

The original published version of this article omitted details of the support provided to the authors’ project, and these details have now been corrected in the online version of the article. The exact change is listed below.

The sentence: “This work was supported by the National Program on Key Basic Research Project (973 Program) of China (2013CB126905)” was changed to “This work was supported by the Ministry of Agriculture of China for Transgenic Research (2014ZX0800938B) and the National Program on Key Basic Research Project (973 Program) of China (2013CB126905)”

