# Supplementary material for: Disruption of OsSEC3A increases the content of salicylic acid and induces plant defense responses in rice
Source: J Exp Bot. 2017 Dec 30;69(5):1051–64. doi: 10.1093/jxb/erx458 (PMC6018903; doi:10.1093/jxb/erx458)
Supplement: Supplementary Figures and Table [file erx458_suppl_supplementary_figures_and_table.pdf]

Disruption of OsSEC3A increases the content of salicylic acid and induces plant defense responses in rice.

Jin Ma, Jun Chen, Min Wang, Yulong Ren, Shuai Wang, Cailin Lei, Zhijun Cheng, and Sodmergn Sodmergn

SUPPLEMENTARY DATA

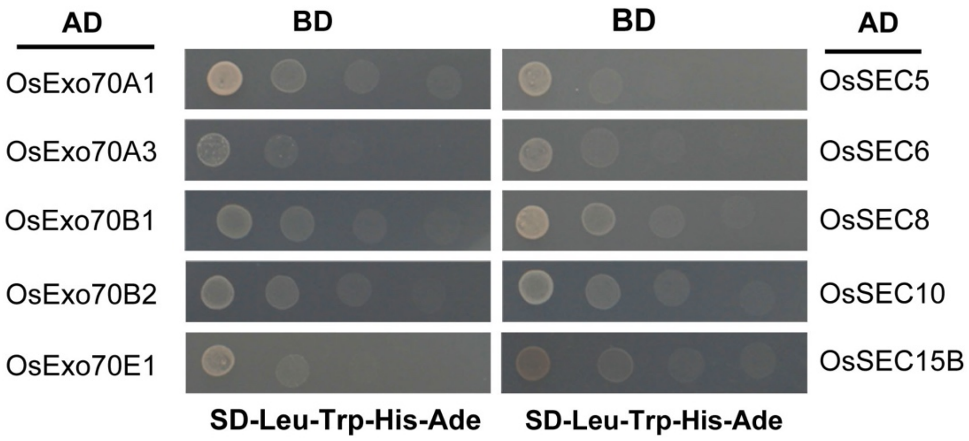

Supplementary Figure S1. Several exocyst subunits fused with AD displayed no auto-activation in our experiments.

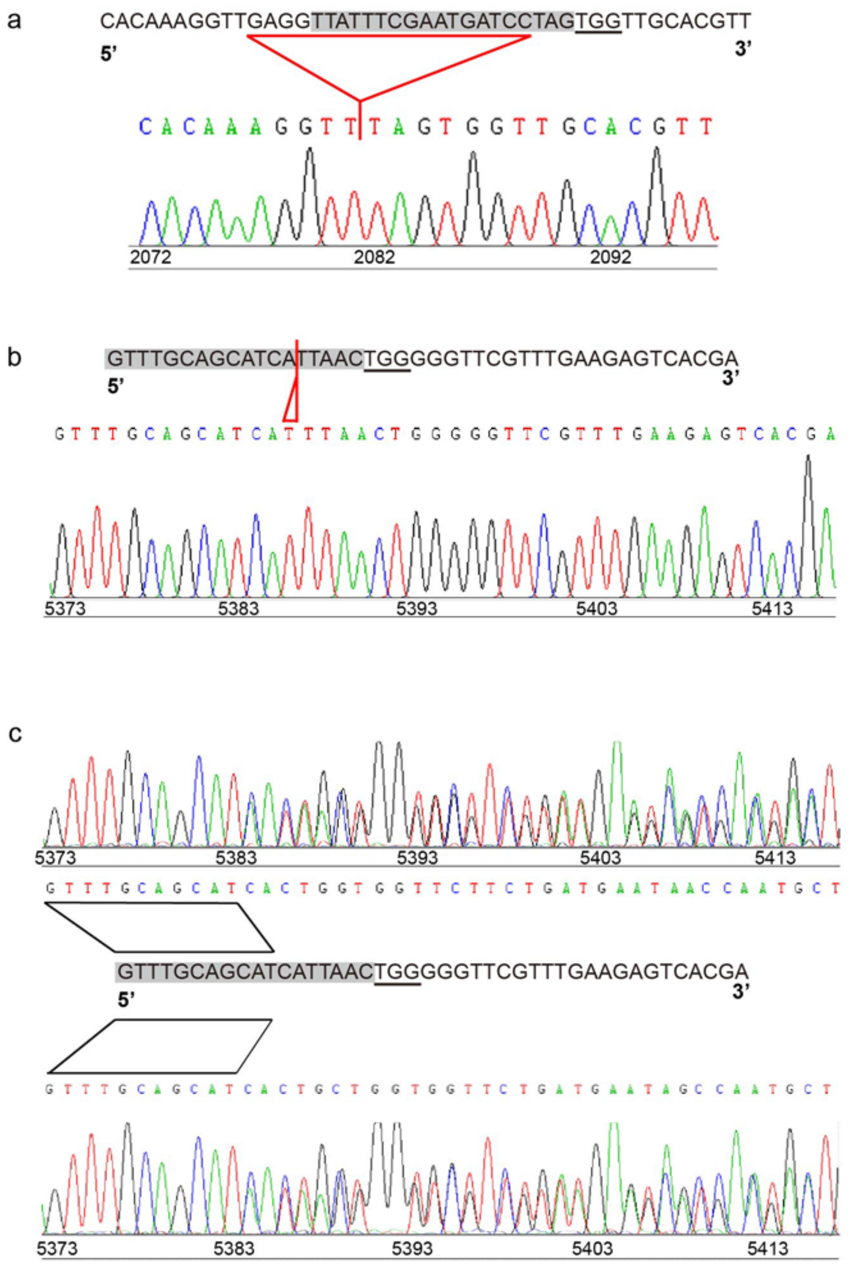

Supplementary Figure S2. Alignment of mutations in *OsSEC3A* from chromatograms to the reference genome sequence. (a-b) Insert-cut indels. (c) Overlapping peaks appear after the presumed cleavage site in both directions, indicating sequence heterogeneity at the site.

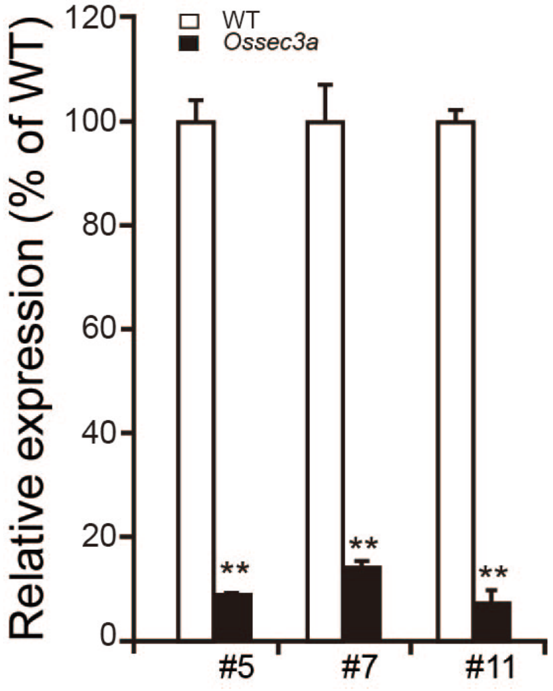

Supplementary Figure S3. qRT-PCR analysis of *OsSEC3A* in WT and *Ossec3a* plants (#5, #7, #11).

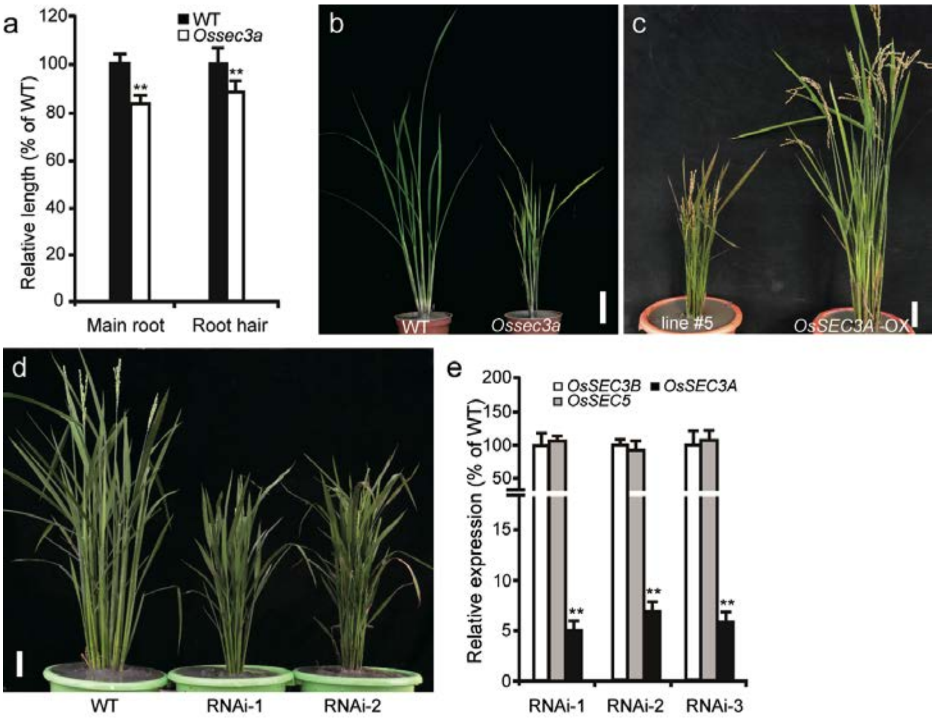

Supplementary Figure S4. Performance of whole plants (WT and transgenic lines) at different developmental stages.

(a) Analysis of root and root hair length in WT and *Ossec3a* plants grown in liquid medium under greenhouse conditions. For all experiments, the longest main roots were observed. (b) Phenotypes of WT and *Ossec3a* mutant plants at the tillering stage. (c) The WT full-length cDNA of *OsSEC3A* completely rescued plant stature. (d) *OsSEC3A*-RNAi transgenic lines mimicked the phenotype of *Ossec3a* mutant plants. Scale bars = 10 cm in (b, c, d). (e) qRT-PCR of *OsSEC3A* and other exocyst subunit genes in WT and *OsSEC3A*-RNAi plants. Data are presented as mean ± standard error (n = 20). Significant differences were identified with Student's *t*-test (**p < 0.01).

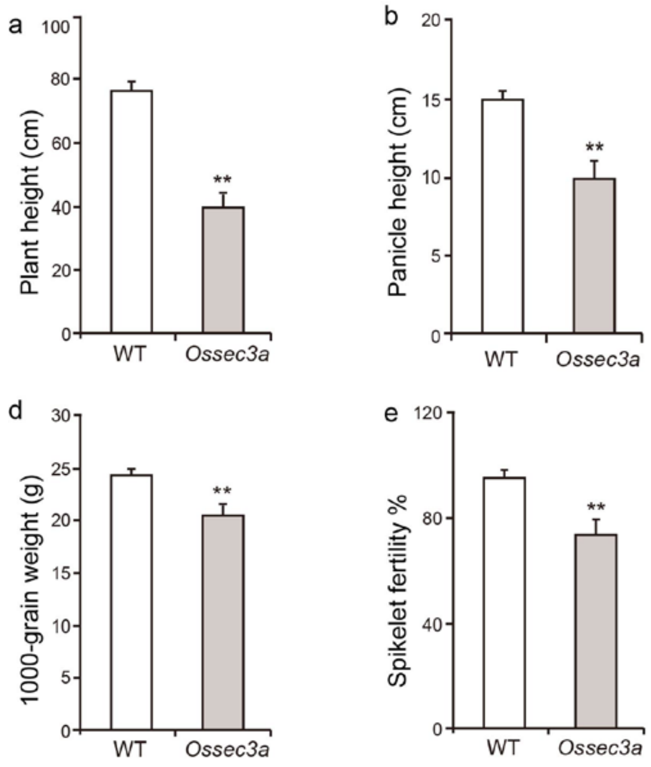

Supplementary Figure S5. Agronomic traits (plant height, panicle length, tiller number, thousand-grain weight, and spikelet fertility) of wild-type and *Ossec3a* mutant plants. Data are presented as mean ± standard error (n = 20). Significance was determined with Student's *t*-test (**$p < 0.01$).

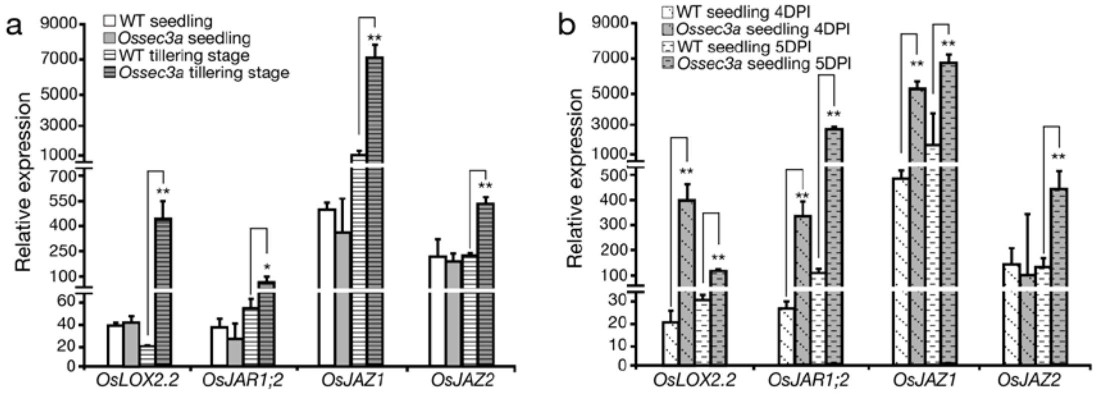

Supplementary Figure S6. qRT-PCR of jasmonic acid (JA) synthesis-related genes (*OsLOX2.2, OsJAR1;2*) and signaling pathway genes (*OsJAZ1, OsJAZ2*) in WT and *Ossec3a* seedling or tillering plants without (a) and with (b) *Magnaporthe oryzae* inoculation. Values are presented as the mean ± standard error of three independent experiments. Significant differences were determined with Student's *t*-test (*0.01 < p <0.05; ** p < 0.01).

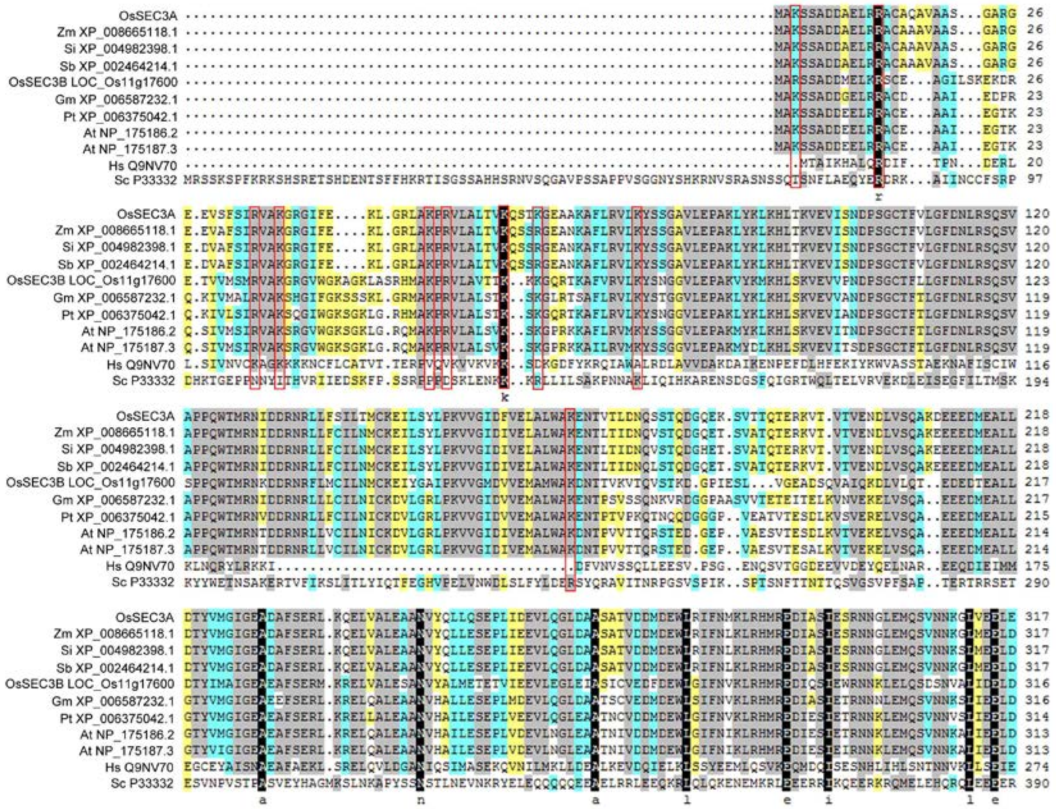

Supplementary Figure S7. Evolutionary analysis of OsSEC3A.

Sequence alignment of OsSEC3A with the equivalent regions of its homologs in different species. Conserved arginine/lysine residues predicted to be important for phosphoinositide binding are highlighted in red. Identical and similar amino acids are shaded in black (100%), grey (>75%), green (50%), or yellow (33%). The following abbreviations were used: Zm, *Zea mays*; Si, *Setaria italic*; Sb, *Sorghum bicolor*; Os, *Oryza sativa*; Gm, *Glycine max*; Pt, *Populus trichocarpa*; At, *Arabidopsis thaliana*; Hs, *Homo sapiens*; Sc, *Saccharomyces cerevisiae*.

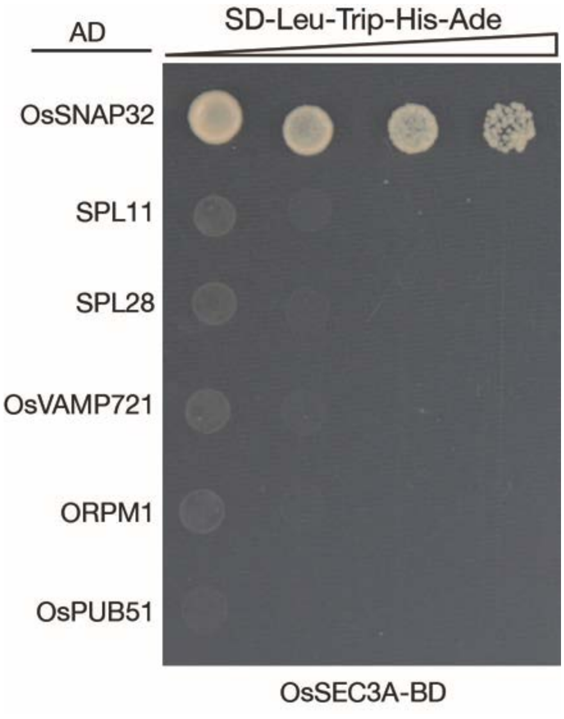

Supplementary Figure S8. Interaction of OsSEC3A with a group of known immunity-and defense-related protein factors. Y2H assay in *S. cerevisiae*. Full-length OsSEC3A was ligated into pGBKT7 to generate the OsSEC3A-BD plasmid. Several exocyst constructs were fused with AD.

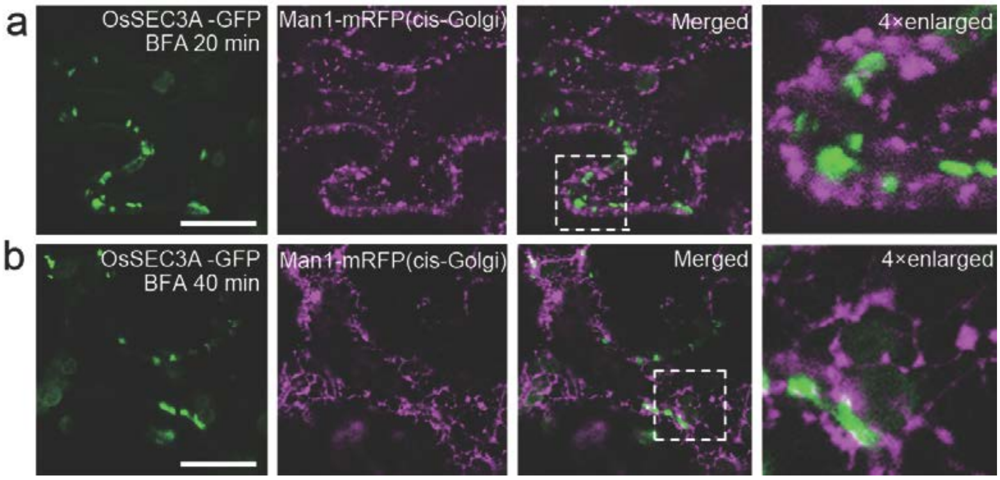

Supplementary Figure S9. BFA treatment of leaf epidermal cells coexpressing OsSEC3A-GFP and Man1-mRF. Scale bars = 10 µm.

Supplementary Table S1. Sequences of DNA oligonucleotides used in this study.

**Primer Name**	**Sequence**
OsSEC3A-Pro-F	CCATGATTAC**GAATTC**CAAGTTTTAGGCA TGGTGTCA
OsSEC3A-Pro-R	CTCAGATCTA**CCATGG**TTGGGGAAGAGG GATGAGTT
OsSEC3A-MBP-F	CGACGGATCC**GAATTC**ATGGCGAAGTCG AGCGCG
OsSEC3A-MBP-R	TACCTGCAGG**GAATTC**CTAGAAGTTAGC AAGGACATC
OsSEC3A ^{N}(a.a. 1–450) -MBP-F	CGACGGATCC**GAATTC**ATGGCGAAGTCG AGCGCG
OsSEC3A ^{N} (a.a. 1–450) -MBP-R	TACCTGCAGG**GAATTC**CTAACAGCTCTTG TCCAGACTC
OsSEC3A ^{C}(a.a. 310–888) -MBP-F	CGACGGATCC**GAATTC**AAAGGACTCGTT GAAGAACT
OsSEC3A ^{C} (a.a.310–888) -MBP-R	TACCTGCAGG**GAATTC**CTAGAAGTTAGC AAGGACAT
qOsCATA-F	CAACCGCAACGTCGACAACTTCTT
qOsCATA-R	TTCACCGGCAGCATCAGGTAGTTT
qOsGSTF10-F	GCTTCTGTGCTCGAAGCCTA
qOsGSTF10-R	AGCAGACGGCATCTTCAGAG
qOsGSTU6-F	TACATCGACGAGGTGTTCCC
qOsGSTU6-R	TGCCTCTGAACACCGGAATC
qOsAOX1a-F	CTTCGCATCGGACATCCATTA
qOsAOX1a-R	TCCTCGGCAGTAGACAAACATC
qOsAOX1b-F	CCTGCTCAGTTCATCACCATCA
qOsAOX1b-R	GCATAAAACGGAGTGACAATAGC
qPO-C1-F	TAGAGGCCGTGTGCAATCAG
qPO-C1-R	GCTTGCACCTGTAGAGTCCC
qEDS1-F	CATTCCAAGAACGAGGACACTG
qEDS1-r	CAAGACTCAAGGCTAGAACCGA
qPAD4-F	AGGGGTTCTTGAGGCTGTGC

qPAD4-R	GCTGAGCTTGACGATGATGTG
qPAL-F	GCACATCTTGGAGGGAAGCT
qPAL-R	GCGCGGATAACCTCAATTTG
qPR1a-F	AGGGCGACTGCAAGCTGGTC
qPR1a-R	ACCACTGCTTCTCCGACACCC
qNPR1-F	GCTGGTGCTCGACTACCTCTACA
qNPR1-R	CAAGGACATCAAGGAGACGC
qPBZ1-F	TGATGGCTCCGGCCTGCGTC
qPBZ1-R	GGTCTTGTATGTGCTTCCCAC
OsLOX2.2-F	CCATCAGTGGACCGACATCA
OsLOX2.2-R	GAGTGGCCGGGTCATTGTCG
OsJAR1;2-F	GGATTGTTGATGGCGATACCT
OsJAR1;2-R	TGCCACCTTTCGTTATGACTTG
OsJAZ1-F	CTGGGACGCTGAAAGACACG
OsJAZ1-R	AACCTCTGCAGCGACACCTT
OsJAZ2-F	CCCCGCTGACCATCTTCTACGA
OsJAZ2-R	GCCTTCTTGTATGGTTCGCTCGT
qOsSEC3A-F	TGTCCCAAGCAAAGGATGAA
qOsSEC3A-R	AAATGCGTAACCACTCATCC
qOsSEC3B-F	TGTGGAGATGGCTATGTGGG
qOsSEC3B-R	GTCTTTCTGAATGGCAACTT
qOsSEC5F-F	CGTGGGAACATTAGAAAAGG
qOsSEC5R-R	CCTCCATTGACTTGTAAAGC
OsSEC3A-GFP-F	GCCCAGATCA**ACTAGT**ATGGCGAAGTCGAGCGC
OsSEC3A-GFP-R	TGCTCACCAT**GGATCC**GAAGTTAGCAAGGACATC
1305OsSEC3A-GFP-F	CGGAGCTAGC**TCTAGA**ATGGCGAAGTCGAGCGC

1305OsSEC3A-GFP-R	TCGAGACGTC**TCTAGA**GAAGTTAGCAAG GACATC
OsSEC3A-Crispr-1F	AGATGATCCGTGGCATTATTTCGAATGATC CTAGGTTTTAGAGCTATGC
OsSEC3A-Crispr-1R	GCATAGCTCTAAAACCTAGGATCATTCGA AATATGCCACGGATCATCT
OsSEC3A-Crispr-2F	AGATGATCCGTGGCAGTTTGCAGCATCAT TAACGTTTTAGAGCTATGC
OsSEC3A-Crispr-2R	GCATAGCTCTAAAACGTTAATGATGCTGC AAACTGCCACGGATCATCT
1390OsSEC3A-CDS-F	TCTGCACTAGGTAC**CTGCAG**ATGGCGAA GTCGAGCGC
1390OsSEC3A-CDS-R	GGGGATCCGTCGAC**CTGCAG**CTAGAAGT TAGCAAGGACATC
OsSEC3A-RNAi1-F	TTACTTCTGCACTA**GGTACC**TGGGCATAG GTGAAGCAGAT
OsSEC3A-RNAi1-R	TAGAGCTCAGGCCT**GGTACC**CGCAAGCG ATCAAGCAACTT
OsSEC3A-RNAi2-F	GAATTCCCGG**GGATCC**TGGGCATAGGTG AAGCAGAT
OsSEC3A-RNAi2-R	CGTAGTCGAC**GGATCC**CGCAAGCGATCA AGCAACTT
OsSEC3A-BD-F	GAATTCCCGG**GGATCC**GTATGGCGAAGT CGAGCGC
OsSEC3A-BD-R	GCAGGTCGAC**GGATCC**CTAGAAGTTAGC AAGGACATC
OsSEC3A (a.a. 1–160)-BD-F	GAATTCCCGG**GGATCC**GTATGGCGAAGT CGAGCGCG
OsSEC3A (a.a. 1–160)-BD-R	GCAGGTCGAC**GGATCC**CTACACAAAATC AATTCCAA
OsSEC3A (a.a. 190–510)-BD-F	GAATTCCCGG**GGATCC**GTCAAACTGAGA GGAAAGTAAC
OsSEC3A (a.a. 190–510)-BD-R	GCAGGTCGAC**GGATCC**CTAAGATACTGT TGAAGTGTCAGC
$OsSEC3A_{C}$ (a.a. 521–888)-BD-F	GAATTCCCGG**GGATCC**GTATCCCACTTCT TGTGGATGA
$OsSEC3A_{C}$ (a.a. 521–888)-BD-R	GCAGGTCGAC**GGATCC**CTAGAAGTTAGC AAGGACATCT
OsSNAP32-AD-F	GGAGGCCAGT**GAATTC**ATGAGCGGGAGG

	AGATCGTT
OsSNAP32-AD-R	CACCCGGGTG**GAATTC**TTATTTTCCAAGC AGACGGCG
OsPUB51-AD-F	GGAGGCCAGT**GAATTC**ATGGAGATCGAG GAGGCGGG
OsPUB51-AD-R	CACCCGGGTG**GAATTC**CTAACTCTTTGTT CTCCAGTC
OsRPM1-AD-F	GGAGGCCAGT**GAATTC**ATGCATACTGAG GTAGGGATCA
OsRPM1-AD-R	CACCCGGGTG**GAATTC**TCATATGGCGGC CGTTCTTT
SPL11-AD-F	GGAGGCCAGT**GAATTC**ATGGCCGGCGAC CGAG
SPL11-AD-R	CACCCGGGTG**GAATTC**TCATACAACCATA GGGTATTGAG
SPL28-AD-F	GGAGGCCAGT**GAATTC**ATGGCGGGCG CGGTGTCGGC GCT
SPL28-AD-R	CACCCGGGTG**GAATTC**TCATATAAGTCTC AGTTCGTAT
OsVAMP721-AD-F	CATCGATACG**GGATCC**ATATGGGGCAGC AGTCGCTGAT
OsVAMP721-AD-R	CGAGCTCGAT**GGATCC**TCACTTGCACTT GAAGCCAT
OsExo70A1-AD-F	CATCGATACG**GGATCC**ATATGGAGACCCT TGCGCAGC
OsExo70A1-AD-R	CGAGCTCGAT**GGATCC**TCAAGTACGCTC TTGTTTTC
OsExo70A3-AD-F	CATCGATACG**GGATCC**ATATGGTGGTGGC GGCGGCGGC
OsExo70A3-AD-R	CGAGCTCGAT**GGATCC**TCAGCGCTTCTG CTCCCCCCAT
OsExo70B1-AD-F	CATCGATACG**GGATCC**ATATGGCGGAGG ACGGCGAGGAGA
OsExo70B1-AD-R	CGAGCTCGAT**GGATCC**TCACTTGGACAC TCCTTCAAAC
OsExo70B2-AD-F	CATCGATACG**GGATCC**ATATGGCTGGCAT GGAGGATACT
OsExo70B2-AD-R	CGAGCTCGAT**GGATCC**TTAGCCTGGGTG GGAGGCAGT
OsExo70E1-AD-F	CATCGATACG**GGATCC**ATATGATGGCTGC TGAGTTAATT
OsExo70E1-AD-R	CGAGCTCGAT**GGATCC**CTACAATGTTTTT TGAGCTCCT

OsSEC5-AD-F	CATCGATACG**GGATCC**ATATGGCGAGCG ACAGCGACGT
OsSEC5-AD-R	CGAGCTCGAT**GGATCC**TCATCGACGCCG CCTCGAG
OsSEC6-AD-F	CATCGATACG**GGATCC**ATATGGAGGATCT GGGCATCGA
OsSEC6-AD-R	CGAGCTCGAT**GGATCC**TTACTGTCCAAG CTTGCTCCAT
OsSEC8-AD-F	GGAGGCCAGT**GAATTC**ATGAGCCGCACC GGCGGCCG
OsSEC8-AD-R	CACCCGGGTG**GAATTC**TTAATGGCCCAA AATCTGAGAG
OsSEC10-AD-F	CATCGATACG**GGATCC**ATATGGAGTTCAA CAGCACACCA
OsSEC10-AD-R	CGAGCTCGAT**GGATCC**CTATTCAGCCATG ATACTGTTT
OsSEC15bN-AD-F	CATCGATACG**GGATCC**ATATGACTGCTCA GACCAAGAAGA
OsSEC15bN-AD-R	CGAGCTCGAT**GGATCC**TCACTTTTTTAGA AGCTTTAGAG
OsSNAP32-GST-F	TGGATCCCCG**GAATTC**ATGAGCGGGAGG AGATCGTT
OsSNAP32-GST-R	GTCGACCCGG**GAATTC**TTATTTTCCAAGC AGACGGCG
OsExo70A1-GST-F	TGGATCCCCG**GAATTC**ATGGAGACCCTT GCGCAGC
OsExo70A1-GST-R	GTCGACCCGG**GAATTC**TCAAGTACGCTC TTGTTTTC
OsExo70B1-GST-F	TGGATCCCCG**GAATTC**ATGGCGGAGGAC GGCGAGGAGA
OsExo70B1-GST-R	GTCGACCCGG**GAATTC**TCACTTGGCACT CCTTCAAAC
NLuc-OsSEC3A$_C$-F	CGGGGGAC**GAGCTC**GGTACCATCCCACT TCTTGTGGATGA
NLuc-OsSEC3A$_C$-R	ACGAGATCTG**GTCGAC**GAAGTTAGCAAG GACATCT
CLuc-OsSNAP32-F	ACGCGTCCCGGGGC**GGTACC**ATGAGCGG GAGGAGATCGTT
CLuc-OsSNAP32-R	AGCTCTGCAG**GTCGAC**TTATTTTCCAAGC AGACGGCG
CLuc-OsSEC5-F	ACGCGTCCCGGGGC**GGTACC**ATGGCGAG CGACAGCGACGT
CLuc-OsSEC5-R	AGCTCTGCAG**GTCGAC**TCATCGACGCCG CCTCGAG

CLuc-OsSEC6-F	ACGCGTCCCGGGGC**GGTACC**ATGGAGGATCTGGGCATCGA
CLuc-OsSEC6-R	AGCTCTGCAG**GTCGAC**TTACTGTCCAAGCTTGCTCCAT
CLuc-OsSEC15bN-F	ACGCGTCCCGGGGC**GGTACC**ATGACTGCTCAGACCAAGAAGA
CLuc-OsSEC15bN-R	AGCTCTGCAG**GTCGAC**TCACTTTTTTAGAAGCTTTAGAG
